# Personalized and Contextualized Persuasion System for Older Adults’ Physical Activity Promoting

**DOI:** 10.1007/978-3-030-51517-1_12

**Published:** 2020-05-31

**Authors:** Houssem Aloulou, Hamdi Aloulou, Bessam Abdulrazak, Ahmed Hadj Kacem

**Affiliations:** 8 Digital Research Centre of Sfax, Sfax, Tunisia; 9 Institut Mines-Télécom, CNRS, Paris, France; 10 Université de Sherbrooke, Sherbrooke, QC Canada; 11 Digital Research Centre of Sfax, Sfax, Tunisia; 12 University of Sfax, Sfax, Tunisia; 13 ReDCAD, University of Sfax, Sfax, Tunisia; 14 ReDCAD, Digital Research Centre of Sfax, Sfax, Tunisia; 15 University of Sherbrooke, Quebec, Canada

**Keywords:** Persuasion strategy, Captology, Physical activities, Older adults, Context-awareness, Semantic modeling, Semantic reasoning, Pervasive technology

## Abstract

Aging often involves a significant change in roles and social positions. The greatest health risk for seniors is the adoption of a sedentary lifestyle that causes isolation, depression, and many diseases. However, convincing an older adult to regularly do physical activities is not generally a simple mission.

This paper proposes a personalized and contextualized persuasion system to promote physical activities for older adults. In fact, our approach considers the personal and health profile of the older adult. It also considers different context parameters (context-awareness). This intelligence is guaranteed thanks to the use of the semantic modeling and reasoning, which from different types of information would be able to decide the best moment to trigger notifications from our persuasive system to the participating older adults.

## Introduction

Nowadays, information technology has a growing influence around the world and brings new opportunities to solve many of society’s problems. One of the most irritating problem is the sedentary lifestyle of older adults. In fact, the lack of physical activities accelerates the transition of people to the age of dependency.

The best way to thwart the sedentary lifestyle among the older adults is to promote physical activities [1]. Generally, older adult trend to limit their physical activities as they often consider them difficult to do. Hence, they must be motivated to change their behavior. Motivation is a set of dynamic factors that guide the action of a person toward a given purpose, which determine his behavior and cause him to behave in a given way or modify his actual behavior [1].

There exist several behaviors change techniques which use persuasion methods to convince subjects to change behaviors. These techniques are inspired by social and cognitive psychology and models associated with them.

We have based our methodology on the output of these technics’ analysis. We have also taken into consideration the older adults’ profiles and contextual information to increase the probability of success of our persuasion technique. Persuasion technics, profiles and contextual information were modeled and realized using the semantic modeling while persuasion strategies were guaranteed using the semantic reasoning.

Following, Sect. 2 presents a detailed state of the art of behavior change persuasion theories and existing systems for promoting physical activities. Section 3 expound our proposed persuasion technique. In Sect. 4, we introduce the used architecture. Then in Sect. 5, we present our first prototype. To validate our persuasive approach and decision-making platform, we propose a first textbook case. Finally, we conclude this article.

## State of the Art

### Behavior Change Persuasion Theories

There are several theories that presents methods that aim to persuade people to change their behavior. Bandura presented a comprehensive theory of human motivation and action called the “Social Cognitive Theory” as an extension of his theory of social learning [2, 3]. In this theory, people are not motivated by internal forces, but by external factors. He emphasizes reciprocal causation through the interplay of cognitive (personal) factors, behavioral factors, and environmental factors. These prominent factors are guided by several variables that intervene in the process of behavior change: self-efficacy, outcome expectations, self-control, reinforcements, emotional coping, and observational learning.

Fogg has identified many principles of persuasion that new technologies can use to influence the behavior of their users. He called this concept the “Captology” (Computers As Persuasive Technology) [4]. He described it as the region where technology and persuasion overlap. For him, behavior change must be voluntary and by conviction. Fogg has also emphasized computer efficacity to persuade users to change behavior through a functional triad. He asserted that, from a user’s point of view, there is three fundamental roles that a computer can play: as tool, as social actor and as media. Each role of the triad is divided into several strategies. For a computer system to be persuasive, it must apply all or most of these strategies.

Additionally, Fogg also demonstrated in his behavior model FBM [5] that 3 conditions must converge at the same time for a behavior to be realized: sufficient “motivation” and “capacity” to do the requested behavior and a well-chosen moment to “trigger” the behavior (physical location, emotions, availability, proximity, etc.) when the person is most open to persuasion (Kairos factor) and thanks to the availability and portability of mobile devices (convenience factor).

For the motivation, Fogg created a framework with three basic motivators formed by tuples in [5]: Sensation (Pleasure/Pain), Anticipation (Hope/Fear) and Belonging (Social acceptance/Social rejection).

Oinas-Kukkonen et al. were inspired by Fogg’s works when proposing the process of “Persuasive System Design” (PSD) [6] that aims to facilitate design and evaluation of persuasive system. The framework is based on seven (7) hypotheses to understand the problems of persuasive systems. The context of persuasion is then analyzed by designers to have a deepening understanding of changes using three (3) major elements: Intention, Event and Strategy. Outcomes are then used to design system’s qualities by meeting several criteria: primary tasks support, dialogue, credibility, and social support.

Later, Oinas-Kukkonen presented the “Behavior Change Support Systems” (BCSS) as an extension of the PSD process [7]. BCSS uses persuasive technology which allows to create, reinforce, and change behaviors. It is based on a design matrix to determine the nature of the behavior change. Rows of the matrix contain types of outcome (formation, altering or reinforcing) and columns contain types of changes (complying, behavior, or attitude).

To help designers in matching target behaviors with solutions for achieving them, Fogg and Hreha proposed the “behavior Wizard” based on the “behavior Grid” [8]. The latter consists of three (3) rows to present the duration of the target behavior and five (5) columns to present the nature of the target behavior. The intersection of a row and a column presents a behavior changing strategy.

Tracking behaviors change of a person involves following the steps of changing actions from the current unwanted behavior to the requested or desired behavior. In this research, we will apply the most important elements of behavior change theories to motivate seniors to adopt a healthy lifestyle. The biggest challenge remaining now is how to adapt the persuasion to the complexity and versatility of every older adult to maximize persuasive effectiveness.

### Existing Systems for Promoting Physical Activity

There are several systems that apply persuasive technology in different domains like health care, leisure, e-commerce, education, etc. Many of these applications were created to promote physical activities. In [9], authors identified 64 apps to promote physical activity among adults. They rated them based on the taxonomy of behavior change techniques. For instance, in [10] research was to understand motivators of changing behavior and sharing results on tweeter by the users of RunKeeper App. It takes the theory of planned behavior as a starting point for their conceptual model. There model consider the influence of altruism, reputation building, community identification, social norms, getting feedback and information sharing. Strava [11] is a persuasive app created for runners and cyclists to track adults activities and offer analysis on their performance. It aims on encouraging running and cycling by competitive motivation when activities are done in group, and challenges when exercising alone. When a participant success the challenge, he earns a “badge” for their “trophy case.” Endomondo [12] is a mobile app that tracks physical activity by monitoring duration, distance and speed. Il motivates users by providing audio feedback, pep talks from friends and user’s friends’ activities and statistics. Flowie is an application that target older adults [13]. In Flowie, the performance of a senior, collected with a pedometer, is translated by the expression of a small animated flower in a touch-screen photo frame. Ubifit Garden [14] and Fish’n’steps [15] are two other applications that use persuasive technology to support healthy behavior via pedometer. Ubifit Garden uses a floral garden wallpaper, in a mobile application, that flourish and blooms as the person performs activities. Move2Play [16] and Healthopia [17] are mobile applications that promote a healthier lifestyle and motivates to participate in regular physical activity using wearable and mobile sensors. Many of the existing systems lack context-awareness when proposing physical activities. They also don’t consider the profile of the person and his health state. There is also no flexibility when asking a person to do behavior nor customization.

## Methodology: Personalization and Customization of the Behavior Change Strategy

We argue that targeting better motivation of senior and successful change of their behavior requires that a persuasion technique that take into consideration, in addition to the Captology elements, several factors in relation with the older adult’s life such as his health state, his environment, his preferred activities and his tendencies. Therefore, a pre-persuasion step is required to form the senior’s profile. The user profile is composed of a personal profile (demographic data, preferred activities and hobbies, social relationships, etc.) and a health profile (physical and mental state). We mainly used the Health Utilities Index Mark 3 (HUI3) [18], a generic health profiles and preference-based systems for measuring health status, reporting health-related quality of life, and producing utility scores, to model the health profile.

**Fig. 1.**
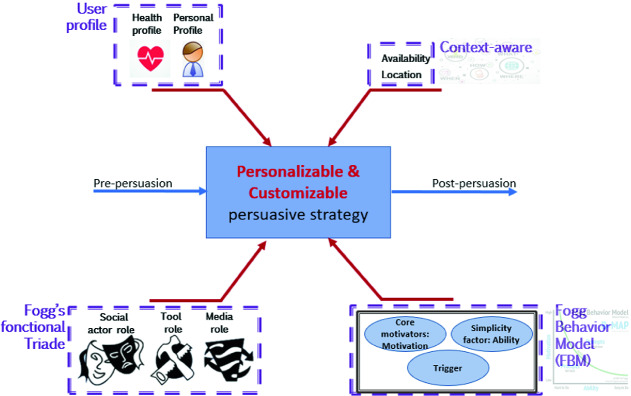
Customizable persuasion strategy

The user profile (personal and health) is then used as an entry to our persuasive strategy in conjunction with contextual information to help increase the likelihood of behavioral change success. Our persuasion strategy inspired from Fogg’s functional triad and Fogg Behavior Model (FBM) uses the received element to convince older adults to adopt a healthy lifestyle and to avoid sedentary. Our approach is presented in Fig. 1 below.

In our persuasion strategy, we took advantage of Fogg’s functional triad and FBM model to promote physical activity of older adults. The following Table 1 presents each element of Fogg’s functional triad and how it is considered in our system.

**Table 1.** Adopted behavior change theory

Role	Implementation	Strategy	Examples
Tool	Provide a mobile app that act as a persuasion tool to convince the older adult to change his behavior	Reduction	Simplify the complexity of activities: giving the difficulty of cleaning the house for an older person, we break this activity down into several sub-activities
		Suggestion	Take into consideration the condition of the user when choosing behavior to change: needs, interests, personality, context of use, environment, proximity, availability and adaptability
		Self-monitoring	The older adult receives information on his performance through the mobile app
Media simulation	The mobile app displays sensitive videos showing dangers of a sedentary lifestyle.		
Social actor	The mobile app will act as a social actor	Physical attractiveness	An attractive GUI with several features to attract the user
		Encouraging language	Notifications on the mobile app have to include positive waves and encouraging language
		Social dynamics	Older adults will feel the need for recognition when the mobile app provides social support and serves them well

We also give great importance to social influence that is based on social comparison to other similar persons, also intrinsic motivation through competition, cooperation, and recognition.

In addition, we will put into practice the Fogg’s behavior model (FBM). We implement the pleasure sensation factor to do a physical activity and the anticipation of hope for a healthy aging. Also, all the 6 factors for maximal ability (time, money, physical effort, brain cycles, social deviance, and non-routine) are considered in our reasoning and persuasion strategy.

## Proposed Architecture

**Fig. 2.**
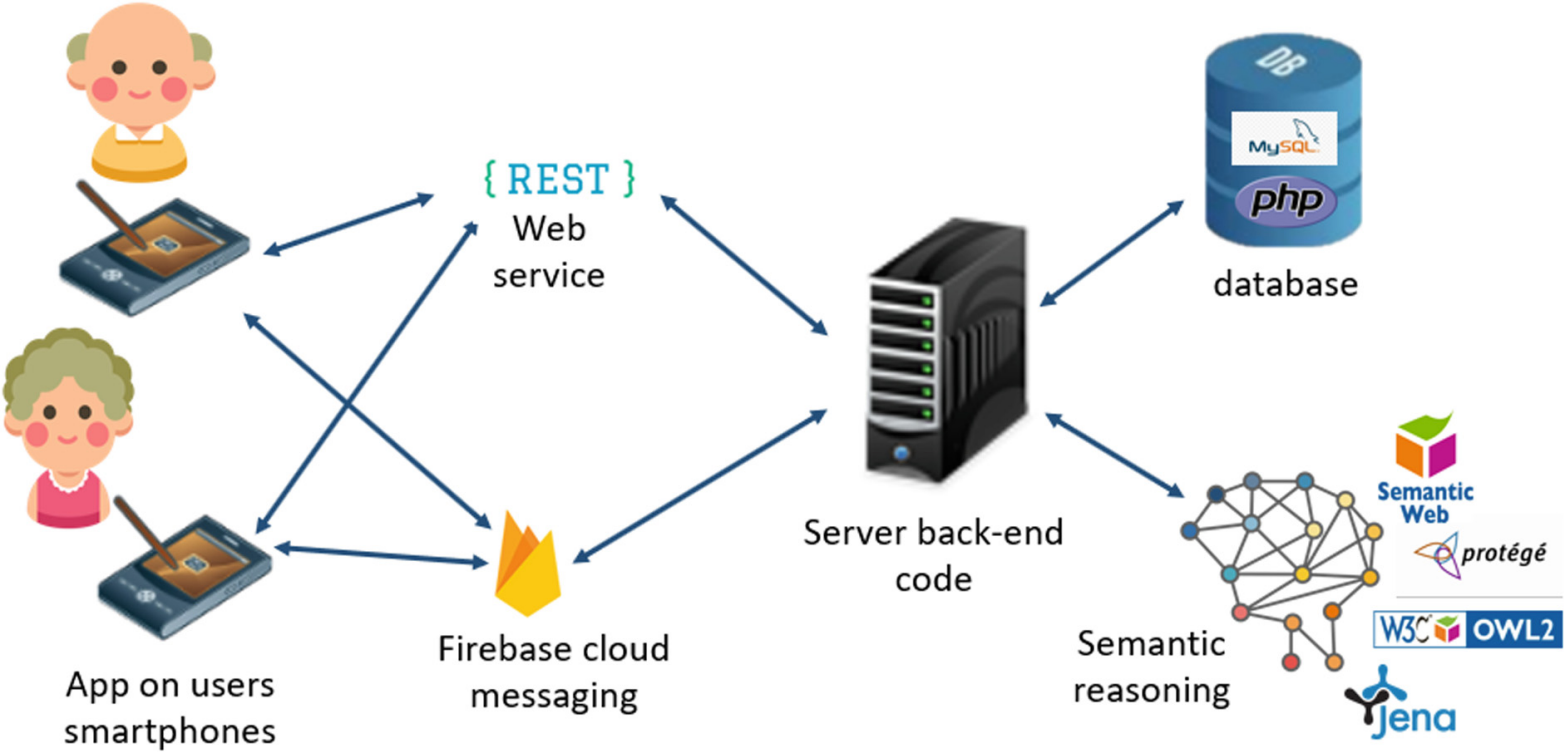
Overview of the architecture using semantic reasoning

The connectivity and portability of smartphones makes possible to reach older adults at any time and place using automated notifications. All notifications and encouragement for an older adult are posted on his smartphone via a mobile app. In addition, this app enables collecting information on senior behavior and sent them instantly to a remote decision-making platform via Internet. The platform filters and processes collected data. Additionally, the reasoning of the context and profile, that is based on an ontological model, allow our platform to choose rationally the best persuasion strategy and to propose, at the right time and place, contextualized physical activities based on several criteria such as his personal profile, his health status, his context, his preferred activities, etc. A typical scenario would be sending a notification requesting to do a given activity only if the older adult has the physical and mental capacity to perform it (based on HUI3 utilities) and considering his context (availability and location). Figure 2 shows a simplified presentation of the architecture of our approach.

## Active Senior’s First Prototype

Our goal at this stage of the project is to develop a computer system serving as proof of concepts that we have named “Active Senior”. The main objective is to validate the functioning of the first components of our system, i.e., the mobile app, the software modules of the server platform and the communication module.

The server app is connected to a database which contains the data of participants, there login information, the list of activities, to which an older adult can register, and the list of notifications received by all participants and information whether the activity has been done or not yet by corresponding person. In fact, when receiving a notification to do a behavior on mobile app, an older adult can inform the server that he accepts to do the behavior and he can inform it when the behavior is done. All information about notifications, the deadline for accepting the activity and carrying it out are stored in the database for statistics purpose.

**Fig. 3.**
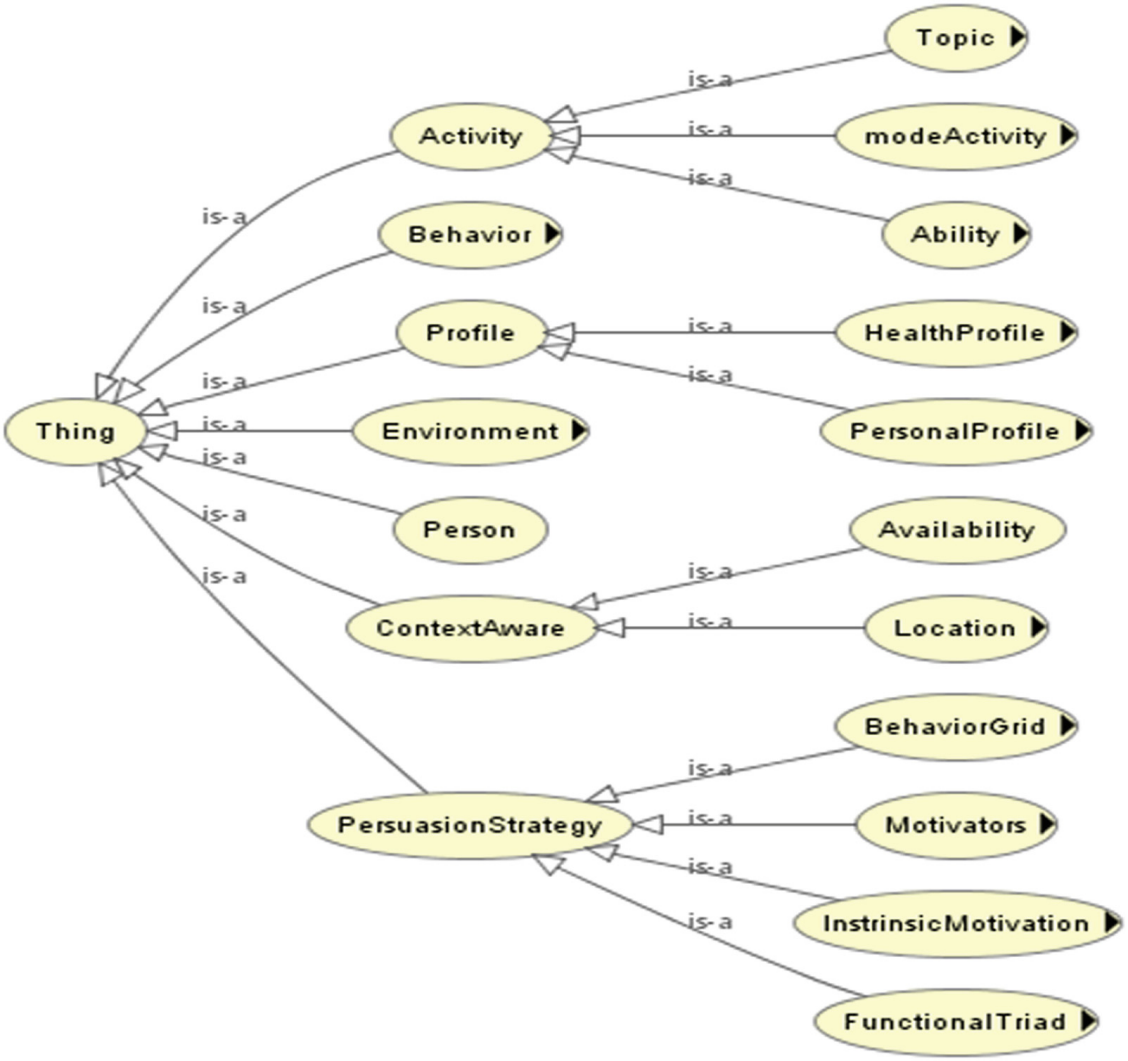
Part of the class hierarchy of our ontology (with OWLViz plugin)

We have defined a new ontology to be able to offer older adults contextualized activities. We have saved our ontology in OWL format which provides a rich vocabulary to add semantics and context and to allow reasoning and inference. Below in Fig. 3 parts of our ontology displayed with OWLViz^1^ plugin. For lack of space, we have chosen to present only the three (3) first levels of our ontological model.

**Fig. 4.**
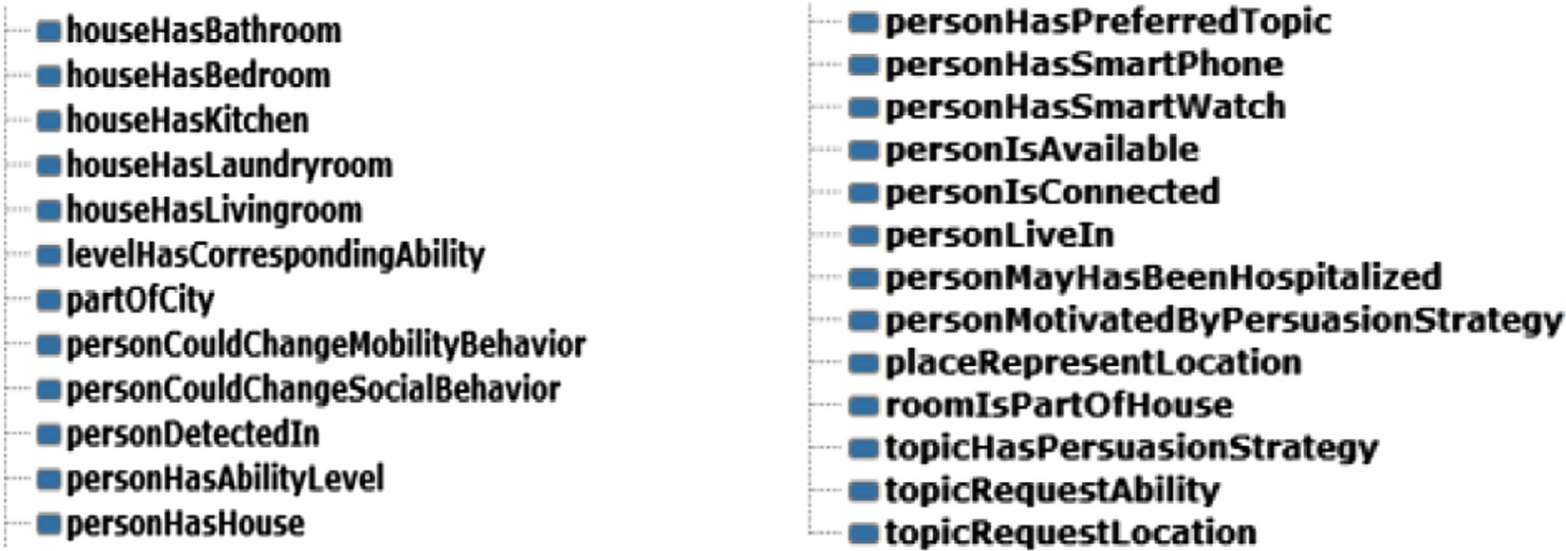
Examples of defined object properties

“Object properties” were defined to describe relationships between two instances of classes (individuals) and “data properties” were defined to describe relationships between instances of classes and their respective values (Fig. 4).

Among created “object properties” we cite:

- “personHasPreferredTopic”: allows to link the “Person” class to the “Topic” class. This will therefore make it possible to know the activities in which the older adult is interested.
- “topicHasPersuasionStrategy”: allows to link a “Topic” to a “PersuasionStrategy.”
- “personIsConnected” and “personIsAvailable”: help to guarantee the person’s context-awareness: his current location and his availability.
- “personMotivatedByPersuasionStrategy”: connects each person to the persuasion strategy that most closely matches his profile, state of health and context.

Among created “data properties” we cite: “persuasionStrategyHasDescription” which allows to assign a description to a “persuasionStrategy” and “levelValue” which have a value between 1 and 6 presenting the value of an instance of the class presenting the HUI3 health indicator for a given person. One of the main benefits of building an ontology-based application is the ability to derive additional knowledge about the concepts modeled by using a reasoner. So, to make our persuasion strategy contextual and personalized, we used the “Apache Jena”^2^ reasoning engine. It is an open source Semantic Web Framework for Java that supports OWL. Thereby, we defined rules so that the reasoner can decide if it would be appropriate to ask a senior to do an activity or not.

The exchanges between the server app and the mobile app are done through REST^3^ (REpresentational State Transfer) web services while the notifications are guaranteed with FCM^4^ (Firebase Cloud Messaging) Google protocol.

**Fig. 5.**
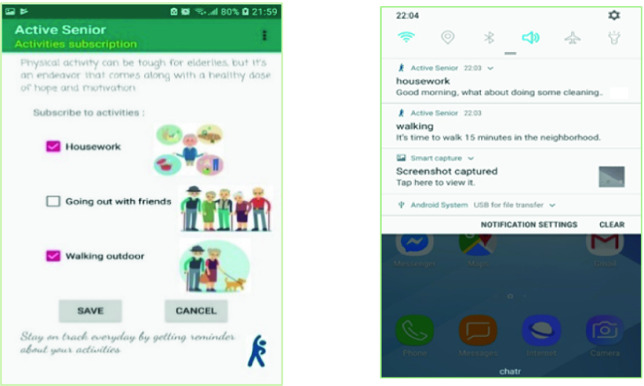
Subscribing/unsubscribing to activities (topics) and receiving notifications

From the mobile app, each senior chooses one or more activities he is interested in. Persuasion of the older adult is done entirely through the mobile app. Through the mobile app, it is possible to:

- Register a new participant: Using web services, login information is added, in real time, to the database on the server.
- Login to a user account: to receive notifications from the server and to be able to communicate with the database.
- Subscribe to one or more activities (topics) by checking in a list the preferred activities. However, it is possible, at any time, to unsubscribe from an activity (by unchecking it in the list). By clicking on the “Save” button, the information is sent in real time to the server and the database is then updated with the user’s new choices. Then, he will no longer receive notifications to do the unchecked activity.
- Receive notifications: When it’s time to do an already chosen activity, the senior receives a notification on his smartphone asking him to do it. We use the self-monitoring technique that we find in several behavior change theories [4]. Indeed, this method empowers the older adult to reach objectives set in advance. While being convinced of the usefulness of the activity to be carried out, the person will make his effort to complete it (Fig. 5).

Our mobile application displays all notifications received by the user and which are incomplete. For each notification, it shows the date and time of its reception, the name of the activity and its description, and 2 buttons:

- Button “Accept”: allows the user to acknowledge receipt and acceptance of the notification. By clicking on this button, the date and time are saved in the database.
- Button “Done”: when the senior complete the requested activity, he clicks on “Done” button. The date and time are sent to the database.

**Fig. 6.**
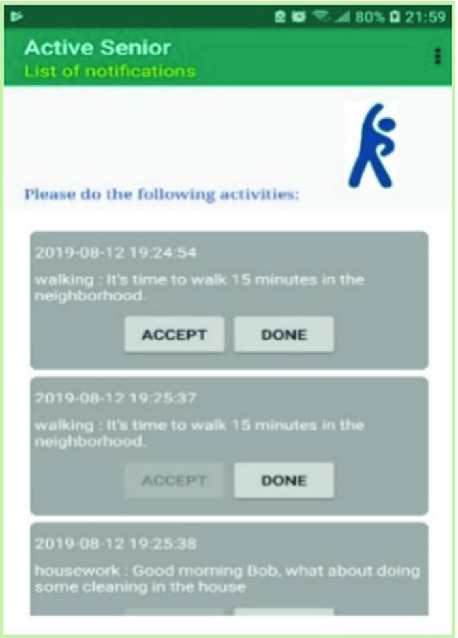
List of notifications received by a senior

The data of the activities carried out by an older adult are saved in the server database for statistical purposes and to periodically encourage participants whose results will experience an evolution. This will motivate seniors to maintain their effort and to try to achieve better performance (Fig. 6).

## Validation

To do a first validation of our approach, we propose a textbook case with a limited number of participants and activities. It is an important step that allows us to test the effectiveness and efficiency of our persuasive approach and our decision-making platform before deploying it on a larger scale.

We have defined three topics (preferred activities): “housework,” “walking outdoor” and “going out with friends”. For each topic we have established some expressions based on Fogg’s motivators such as “encouraging language”, “hope anticipation” and “fear anticipation”. For instance, a notification to do housework that uses a hope anticipation motivator may contains the text “A clean and organized home is a beautiful home. It eliminates the risk of developing allergies and asthma; it also reduces stress and it is extremely vital to your mental health”. A fear anticipation motivator in a notification to walk outdoor can be done with the text “Sedentary lifestyle can lead to difficulties in performing the activities of daily living, there’s greater risk of heart disease, diabetes and depression.” An encouraging language motivator in a notification to go out with friends can be “An outing is scheduled tonight by your kind friends. It will be a good opportunity to relax, exchange stories and forget everyday worries”.

We have also created the profiles of three older adults: “JohnDoe”, “JohnWalker” and “JohnGoe”. Each profile contains the physical and health status of the person, his concerns, his environment, his availability, and his location. “JohnDoe” is subscribed to “housework” activity, “JohnWalker” is subscribed to “walking outdoor” activity and “JohnGoe” is subscribed to “going out with friends” activity.

**Fig. 7.**
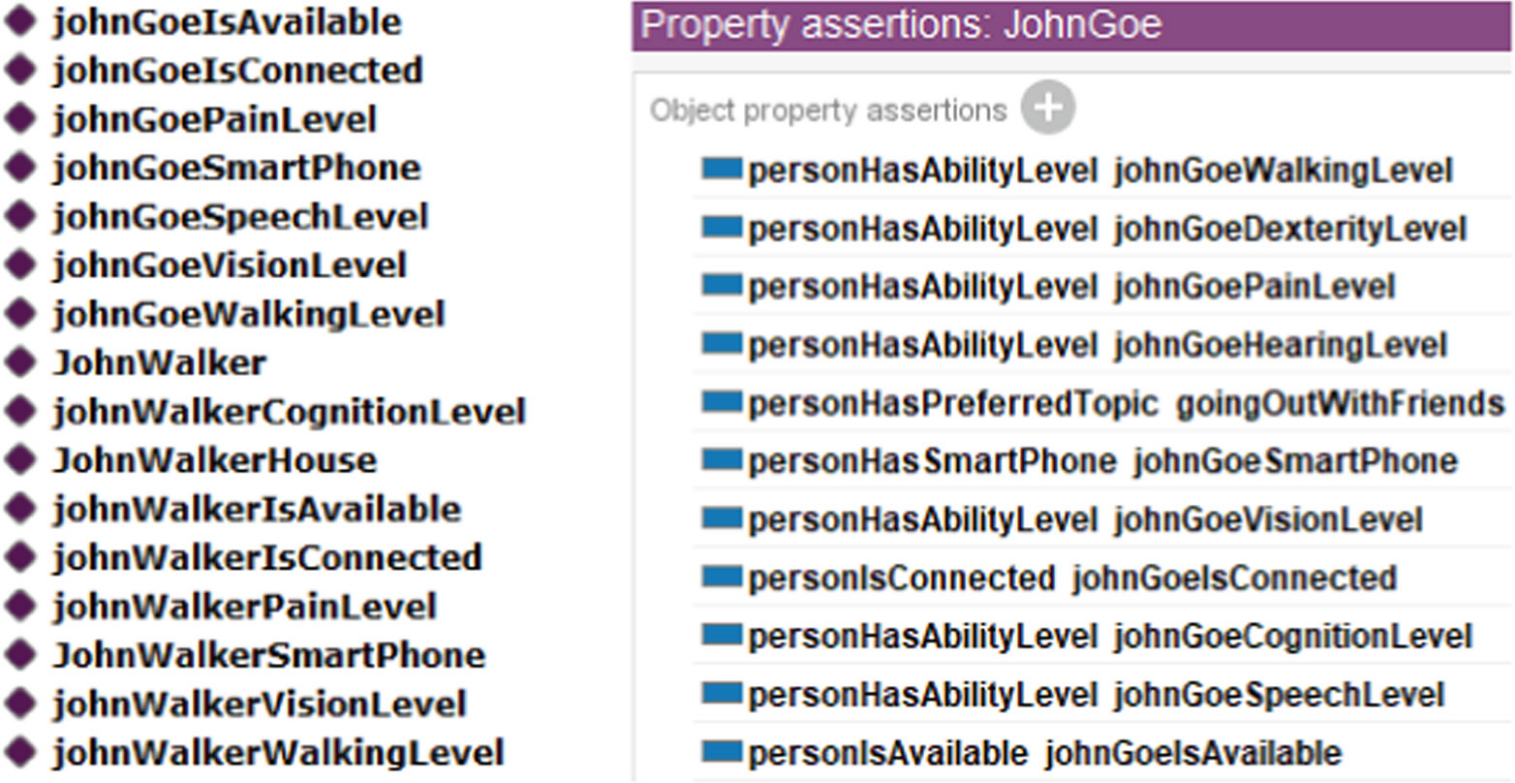
Some older adults’ individuals and property assertions from the ontology

The ontology allows to personalize and customize the persuasive strategy according to the older adult’s profile. In Fig. 7 below, we have some Individuals defined in the ontology for the participating older adults and “Property assertions” used for “JohnGoe”.

Using the ontology, it is possible to generate new knowledge using inference rules. In fact, inference rules allow to decide the best moment to trigger a behavior.

Here is an example of a used rule written in Jena syntax:
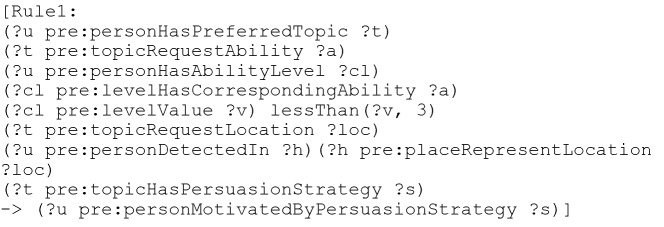


Using this rule, the person is motivated by the persuasion strategy only if the following conditions are valid:

- The older adult has a favorite topic and the topic has a persuasion strategy.
- The chosen topic requires one or more health capacities among the 8 elements of HUI3 health status (Vision, Hearing, Speech, Ambulation, Dexterity, Emotion, Cognition, Pain).
- For each state of health, the person has a level between 1 and 6. When the value is 1, it means that he has no health problems for the state of health.
- It is not possible to allow the older adult to do the activity if the value of the health level is > 3 because it could present a risk to his safety and health.
- We also check if the topic requests a location and if the person is this location.

Our persuasion strategy is therefore personalized in the sense that it respects abilities, habits, choices, health situation, context, etc. of the person being followed.

**Fig. 8.**
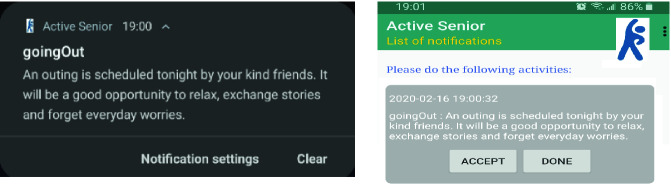
Received notification by JohnGoe

Figure 8 below show a notification received by JohnGoe whose profile meets the conditions of the ontology rule mentioned above.

## Conclusion

Persuasive technologies have shown a lot of potentials to influence the behavior and attitudes of individuals. In this paper, we adopted Fogg’s persuasive techniques to promote physical activity of older adults. To change the behavior of an older adult, we defined a realistic persuasion strategy which considers senior’s profile, his environment and his context. To make our persuasion strategy customizable and contextualizable, we defined an ontology and used rules to deduce new knowledges in order to choose the right moment and place to request the execution of an activity. As first validation of our approach, we developed a first prototype of “Active Senior” to test the used concepts. The first results seem to be promising.

To be successful, new behavior must be maintained and preserved as several older adults may adopt the new proposed behavior for a limited period and they give up after a certain time. This can be explained by the fact that preserving a behavior or attitude requires a lot of effort, energy and time, etc.

In future work, our work will be validated with real volunteer subjects who will be asked to use the mobile app to promote their physical activities. Selection criteria will be set in order to choose subjects with different profiles (physical capacity, habits, social situation, health status, environment, etc.). This will allow us to validate the proposed solution with different profiles.

## Footnotes

1 https://github.com/protegeproject/owlviz.

2 https://jena.apache.org/.

3 https://restfulapi.net/.

4 https://firebase.google.com/docs/cloud-messaging.
